# Associations of HIV testing and late diagnosis at a Japanese university hospital

**DOI:** 10.6061/clinics/2016(02)04

**Published:** 2016-02

**Authors:** Tetsuya Horino, Fumiya Sato, Tetsuro Kato, Yumiko Hosaka, Akihiro Shimizu, Shinji Kawano, Tokio Hoshina, Kazuhiko Nakaharai, Yasushi Nakazawa, Koji Yoshikawa, Masaki Yoshida, Seiji Hori

**Affiliations:** Jikei University School of Medicine, Department of Infectious Diseases and Infection Control, Minato-ku, Tokyo, Japan

**Keywords:** HIV Testing, Late Diagnosis, Voluntary HIV Testing, Hypergammaglobulinemia, Thrombocytopenia

## Abstract

**OBJECTIVES:**

This study was conducted to clarify the rate of late diagnosis of HIV infection and to identify relationships between the reasons for HIV testing and a late diagnosis.

**METHODS:**

This retrospective cohort study was conducted among HIV-positive patients at the Jikei University Hospital between 2001 and 2014. Patient characteristics from medical records, including age, sex, sexuality, the reason for HIV testing and the number of CD4-positive lymphocytes at HIV diagnosis, were assessed.

**RESULTS:**

A total of 459 patients (men, n=437; 95.2%) were included in this study and the median age at HIV diagnosis was 36 years (range, 18–71 years). Late (CD4 cell count <350/mm^3^) and very late (CD4 cell count <200/mm^3^) diagnoses were observed in 61.4% (282/459) and 36.6% (168/459) of patients, respectively. The most common reason for HIV diagnosis was voluntary testing (38.6%, 177/459 patients), followed by AIDS-defining illness (18.3%, 84/459 patients). Multivariate analysis revealed a significant association of voluntary HIV testing with non-late and non-very-late diagnoses and there was a high proportion of AIDS-defining illness in the late and very late diagnosis groups compared with other groups. Men who have sex with men was a relative factor for non-late diagnosis, whereas nonspecific abnormal blood test results, such as hypergammaglobulinemia and thrombocytopenia, were risk factors for very late diagnosis.

**CONCLUSIONS:**

Voluntary HIV testing should be encouraged and physicians should screen all patients who have symptoms or signs and particularly hypergammaglobulinemia and thrombocytopenia, that may nonspecifically indicate HIV infection.

## INTRODUCTION

With the development of antiretroviral therapy (ART), the mortality and morbidity associated with HIV have decreased 1. In particular, Lohse et al. demonstrated an estimated median survival of >35 years for a young HIV-positive individual 2. Previous studies have also shown that survival improved with early initiation of ART 3,4 and that ART initiation at CD4 counts <500/mm^3^ improved the course of HIV infection 5. In contrast, late diagnosis and delayed treatment of HIV infection contribute to the development of opportunistic infections, such as pneumocystis pneumonia and cytomegalovirus infection and increase hospitalization and mortality risks 6,7,8. Moreover, reduction of HIV viral load by early initiation of ART decreases the transmission rate of HIV infection 9. Therefore, a World Health Organization guideline strongly recommends ART initiation for HIV patients when CD4 counts are <500/mm^3^.

Timely initiation of ART requires an early diagnosis of HIV infection, which can also contribute to reducing the prevalence of high-risk sexual behavior conducive to HIV transmission 10. Thus, early diagnosis of HIV infection is crucial to improve patient survival and decrease the transmission rate. Although various organizations recommend HIV testing for early diagnosis, many patients are only diagnosed with HIV infection when they develop AIDS-defined illness. An annual report showed that more than 1,000 people have been newly diagnosed with HIV infection in Japan since 2007 and that 24,561 patients had been diagnosed with HIV infection by 2014. In 2014, 29.4% (455/1,546 patients with newly diagnosed HIV infection) of patients were diagnosed with HIV infection upon presenting with an AIDS-defining illness (http://api-net.jfap.or.jp/status/2014/14nenpo/h26gaiyo.pdf). The rate of late diagnosis of HIV infection differs among various countries. Hall et al. demonstrated that the highest percentage of people diagnosed with late-stage disease was among people in the United States (28.7%), followed by Australia (18.8%), France (15.3%), Italy (14.5%) and Canada (8.8%) 11. In the present study, we evaluated the reasons for HIV testing and their relationships with a late diagnosis of HIV infection.

## MATERIALS AND METHODS

### Study population

The present study was conducted at a 1,075-bed hospital in Tokyo, Japan, affiliated with the Jikei University School of Medicine, which is one of the AIDS treatment core hospitals. The study included adult patients with HIV infection who visited the hospital between January 2001 and December 2014.

### Study design

A retrospective cohort study was conducted to investigate the reasons for HIV testing and their relationships with a late diagnosis of HIV infection. Patient characteristics, such as age, sex, sexuality, the reason for HIV testing and the CD4-positive lymphocyte count at HIV infection diagnosis, were assessed from the patients' medical records at the hospital.

**Figure 1:**
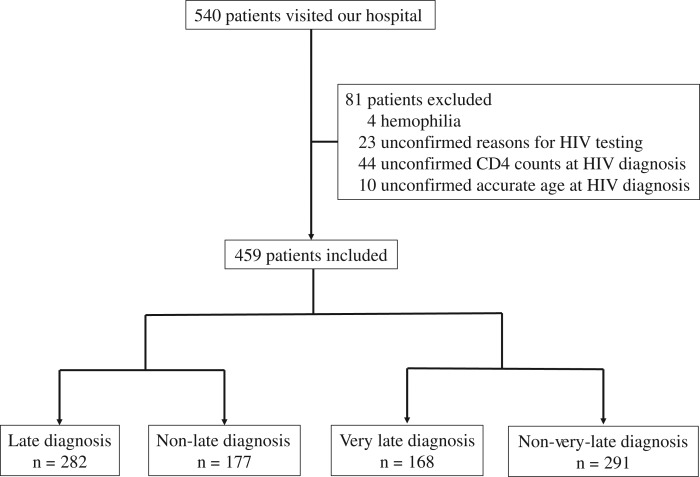
Chart of the inclusion and exclusion criteria in this study.

The reasons for HIV testing were classified into the following six categories: AIDS-defining illness, sexually transmitted infection (STI), nonspecific symptoms, nonspecific abnormal blood test results, routine testing and voluntary testing. Patients with AIDS-defining illness were those diagnosed with at least one of the diseases listed by the Centers for Disease Control and Prevention. Moreover, genital herpes was considered an AIDS-defining illness, regardless of whether it was the first or a repeated episode. STIs included syphilis, amebic disease, hepatitis B, urethritis and condyloma acuminatum. Nonspecific symptoms were defined as those that were not associated with an AIDS-defining illness or STI. Routine testing included examination before a medical procedure, such as blood donation, hospitalization, or an operation. The date of the positive HIV test was the earliest date of a documented positive anti-HIV antibody test from a laboratory report at our hospital or another medical institute, including other hospitals, clinics and healthcare centers. Late diagnosis and very late diagnosis were defined as CD4-positive T lymphocyte counts <350 cells/mm^3^ and <200 cells/mm^3^, respectively, at the time of the initially positive HIV test 12. We excluded patients with hemophilia because we could not determine when they were infected through blood products. Furthermore, patients for whom the reason for HIV testing, the CD4 count and an accurate age at the time of HIV diagnosis could not be confirmed were excluded from this study (Figure 1).

### Statistical analyses

The chi-square test or Fisher's exact test was used to compare categorical variables. The Mann-Whitney U test was used to compare continuous variables. To determine relative factors for late and very late diagnosis, a multiple logistic regression model was used to control for potential confounding variables. We present the results of the logistic regression analysis as an adjusted odds ratio (AOR) with a 95% confidence interval (CI). *P* values were two-tailed and statistical significance was set at *p*<0.05. These statistical analyses were conducted using SPSS version 19 (IBM Japan Ltd).

### Ethics

We could not obtain informed consent from the patients because the present study was retrospective cohort study. Therefore, we posted an announcement about this study. This study does not present data that identify the patients and was approved (approval no. 26-184 (7689)) by the institutional ethics committee of the Jikei University Hospital.

## RESULTS

### Patient characteristics

**Figure 2:**
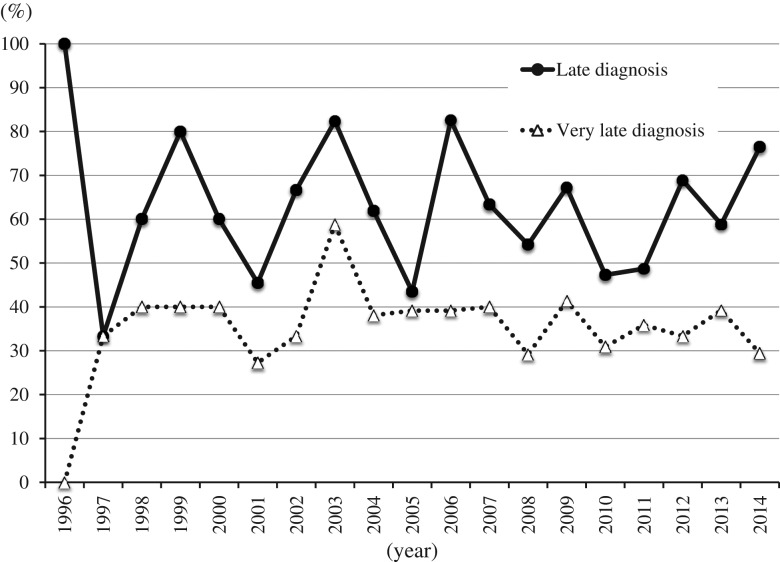
Proportions of late and very late diagnoses in this study. The rates of the two diagnoses were similar during the study period.

A total of 540 patients infected with HIV visited our hospital during the investigation period. Among these patients, 81 were excluded from this investigation; 4 of these patients had hemophilia and we could not confirm the reason for HIV testing for 23 patients, CD4 counts for 44 patients, or an accurate age at the time of HIV diagnosis for 10 patients. Therefore, in total, 459 patients met the inclusion criteria of this study (Figure 1). HIV diagnosis occurred between 1996 and 2014 for all subjects, of whom 437 (95.2%) were men and the median age was 36 (range, 18–71). Of the 437 male subjects, 359 patients (82.2%) were men who have sex with men (MSM) (Table 1). The median CD4 count at diagnosis was 283/mm^3^ (range, 4–1443 mm^3^). A total of 282 patients (61.4%) and 168 patients (36.6%) of the 459 subjects were categorized as having a late or very late diagnosis, respectively, and their rates remained approximately the same during the study period (Figure 2).

### Reasons for HIV testing

As shown in Table 1, the most common reason for HIV testing was voluntary testing (38.6%, 177/459), followed by AIDS-defining illness (18.3%, 84/459 patients). Among AIDS-defining illnesses, *Pneumocystis jirovecii* pneumonia (PCP) was the most common (59.5%, 50/84 patients) disease associated with HIV infection, followed by Kaposi's sarcoma (8.3%, 7/84 patients). Other patients were found to have HIV infection due to the following AIDS-defining illnesses: candidiasis of the esophagus, 3 patients; cryptococcal meningitis, 2; cryptosporidiosis, 1; cytomegalovirus infection, 4; HIV-related encephalopathy, 3; herpes simplex, 3; pulmonary tuberculosis, 3; lymphoma, 4; progressive multifocal leukoencephalopathy, 1; and toxoplasmosis of the brain, 1. Two patients with multiple AIDS-defining illnesses underwent HIV testing because they had PCP and esophageal candidiasis.

However, 53 of the 459 patients (11.5%) tested positive for HIV when they were diagnosed with other STIs. The most common STI was syphilis (37.7%, 20/53 patients), followed by amebic disease, condyloma acuminatum and hepatitis B (each 18.9%, 10/53 patients). Of the 2 patients with urethritis, one had urethritis caused by *Neisseria gonorrhoeae* infection and the other had non-gonococcal and non-chlamydial urethritis. A patient with multiple STIs was tested for HIV because he had a past history of hepatitis B and syphilis.

In total, 95 patients (20.7%) were diagnosed with HIV infection as one of the differential diagnoses for nonspecific symptoms or nonspecific abnormal blood test results not classified as definitive for an AIDS-defining illness or STIs. Among 80 patients who had nonspecific symptoms, 11 (13.8%) were tested for the anti-HIV-1 antibody due to fever without any symptoms. The most common nonspecific symptom other than fever was skin lesions (26.3%, 21/80 patients), including nonspecific skin eruption, 11 patients; herpes zoster, 6; molluscum contagiosum, 2; erythema multiforme, 1; and scabies, 1. Lymphadenopathy was also a common symptom leading to HIV testing (12.5%, 10/81 patients), including cervical lymphadenopathy in 8 patients, generalized lymphadenopathy in 1 patient, and inguinal lymphadenopathy in 1 patient. Two patients were tested for HIV when they had other infectious diseases; one patient had nonrecurrent bacterial pneumonia and the other had candidemia because of a catheter-related bloodstream infection. Five patients had multiple symptoms when they were diagnosed, including lymphadenopathy and skin rash (n=3), oral candidiasis and lymphadenopathy (n=1) and oral candidiasis and skin rash (n=1). Eleven patients, including those who had multiple symptoms, were tested for HIV because of oral candidiasis. Among these 11 patients with oral candidiasis, 11 patients (100%) were diagnosed with HIV infection as a late diagnosis and 9 patients (81.8%) received a very late diagnosis.

Fifteen patients were tested for anti-HIV antibody to more closely examine their abnormal blood test results. The most common abnormal result was hypergammaglobulinemia (46.7%, 7/15 patients), followed by thrombocytopenia (26.7%, 4/15 patients), pancytopenia (13.3%, 2/15 patients) and hemophagocytosis (6.7%, 1/15 patients). One patient was tested for HIV because he had hypergammaglobulinemia and thrombocytopenia. All 8 patients with hypergammaglobulinemia and 5 of 7 patients (71.4%) with thrombocytopenia were categorized as having a late diagnosis. Moreover, 7 of 8 patients (87.5%) hypergammaglobulinemia and 4 of 7 patients (57.1%) with thrombocytopenia received a very late diagnosis.

In addition, 50 patients were diagnosed with HIV infection by routine testing. The most common reason for routine testing was evaluation prior to a medical procedure (72.0%, 36/50 patients), including an operation (26), endoscopy 5, hospitalization 2, fertility treatment 2, or a clinical trial 1. Additionally, 12 patients (24.0%) were found to be HIV positive on testing prior to blood donation and two patients were diagnosed as HIV positive during prenatal care.

### Relationships between late and very late diagnosis and the reason for HIV testing

As shown in Table 1, in univariate analysis, patients in the late diagnosis and very late diagnosis groups were significantly older than those in the other groups. In addition, AIDS-defining illness and nonspecific abnormal blood test results were significantly more common as reasons for HIV testing in the late and very late diagnosis groups than in the other groups, whereas MSM and voluntary testing were significantly less common reasons. In addition, STI was a factor associated with very late diagnosis. Multivariate analysis using the logistic regression model revealed independent predictive factors for late diagnosis, which included AIDS-defining illness (AOR=11.410; 95% CI=3.998–32.563; *p*<0.001), whereas the rates of MSM and voluntary testing in non-late diagnosis were significantly higher than those in late diagnosis (AOR=0.488; 95% CI=0.287–0.831; *p*=0.008 and AOR=0.418; 95% CI=0.274–0.638; *p*<0.001, respectively) (Table 2a). In contrast, AIDS-defining illness and nonspecific abnormal blood test results were found to be independent relative factors for very late diagnosis (AOR=11.196; 95% CI=5.747–21.814; *p*<0.001 and AOR=5.637; 95% CI=1.723–18.443; *p*=0.004, respectively), whereas voluntary testing was significantly less frequent among patients with very late diagnosis in the logistic regression analysis (AOR=0.353; 95% CI=0.210–0.593; *p*<0.001) (Table 2b).

**Table 2b:** 

	Adjusted odds ratio	95% CI	*p*-value
AIDS-defining illness	11.196	5.747–21.814	<0.001
Voluntary testing	0.353	0.210–0.593	<0.001
Nonspecific abnormal result	5.637	1.723–18.443	0.004

## DISCUSSION

In Japan, people can choose to undergo HIV testing at any medical facility, including healthcare centers, clinics and hospitals. In addition, several of these facilities provide voluntary counseling and testing for HIV that are free and anonymous. However, after identification of HIV infection, most HIV patients have to transfer to an AIDS treatment core hospital, which can treat patients with HIV infection or opportunistic infections. Not all of these AIDS treatment core hospitals have all medical care equipment for specific situations, such as deliveriesy, pediatric care or tuberculosis treatment. Therefore, these patients have to transfer to other AIDS treatment core hospitals that have adequate facilities. Patients with HIV infection are usually treated with ART based on the Japanese guideline, which is similar to other guidelines, such as United States Department of Health and Human Services guideline. Indeed, for asymptomatic HIV patients, the timing of ART initiation depends on the CD4 count, with the cut-off being less than 200/μL until 2006, less than 350/μL from 2006–2013 and less than 500/μL since 2013. HIV patients can receive medical care at public expense; the expense that the patient pays depends on his or her income, with patients responsible for paying 0–10% of the cost (less than 20,000 yen) of medical practice, including examination and ART. Therefore, we do not believe that cost causes hesitation to undergo HIV testing in Japan.

Most previous studies have defined CD4 cell counts <200 cells/mm^3^ as indicative of late HIV diagnosis. However, Kitahata et al. showed that the mortality rate in patients starting ART at CD4 counts <350 cells/mm^3^ is significantly higher than that in patients starting ART at CD4 counts of 350–500 cells/mm^3^ 3. Consequently, current guidelines strongly recommend starting ART at CD4 counts <500 cells/mm^3^. The European Late Presenter Consensus Working Group defined late presentation as CD4-positive T lymphocytes <350 cells/mm^3^ or as presentation with an AIDS-defining illness, regardless of the CD4 cell count and defined presentation with advanced HIV disease as <200 cells/mm^3^ or presentation with AIDS-defining illness, regardless of the CD4 cell count 13. In the present study, we defined late diagnosis as a CD4-positive T lymphocyte count <350 cells/mm^3^ and very late diagnosis as a count <200 cells/mm^3^ at diagnosis, excluding AIDS-defining illness, regardless of the CD4 cell count. This was a similar approach to that reported in another study 12 because we included AIDS-defining illness as one of the reasons for HIV testing in the current study.

Other studies demonstrated that the rate of very late diagnosis was 8.8–43% 7,11,12,14 and differed among countries. In our study, we demonstrated that the rate of very late diagnosis was 36.6%, which was similar to results from Canada (39%) and Italy (39%) 7. In contrast, the rate of late diagnosis has been reported as 51.3–63.5% 12,14,15, which is similar to our result, or 61.4%. Girardi et al. demonstrated that MSM was the factor responsible for the reduced prevalence of late diagnosis 7. In our study, we found that that MSM was a relatively associated factor for non-late and non-very-late diagnosis; however, this finding for non-very-late diagnosis was not supported in the multivariate analysis. These results suggest that MSM was a factor promoting HIV testing for both patients and physicians.

In our study, 80 of 459 patients (17.4%) were diagnosed with HIV infection because they had nonspecific symptoms. Common symptoms in patients with primary HIV infection included pharyngitis, lymphadenopathy and skin eruptions 14. Certain patients with these symptoms were diagnosed with HIV infection in the acute phase, although we could not determine the accurate rate of primary HIV infection in our patients. Eleven patients, including patients who had multiple symptoms, were diagnosed with HIV infection because of oral candidiasis on testing. In this study, oral candidiasis was classified as a nonspecific symptom because we aimed to evaluate the symptom as a reason for HIV testing. However, all patients and 9 of 11 patients were categorized as having a late or very late diagnosis, respectively. Therefore, we infer that an immunocompromised status should be suspected and that HIV testing should be conducted immediately in these patients in the absence of other risk factors, such as inhalational steroid use. In addition, we determined that certain patients were found to be HIV positive as part of the differential diagnosis of meningitis, weight loss, or a fever of unknown origin. Wohlgemut et al. demonstrated that CD4 counts in patients who were not diagnosed at first presentation were frequently < 200/mm^3^ compared with the counts of those who were diagnosed at first presentation 12. Thus, these results indicate that HIV screening should be initiated without hesitation upon the first suspicion of viral infection or symptoms unexplained by other diseases.

In the present study, 15 patients were tested for anti-HIV antibody to more closely examine their abnormal blood test results. Because these abnormal results, including hemophagocytosis, improved after ART, we determined that the abnormal results were because of HIV infection. The incidence of monoclonal gammopathy of undetermined significance (MGUS, or benign monoclonal gammopathy) is 3–26% in HIV patients 15, so certain patients with HIV infection have hypergammaglobulinemia, although the causal mechanism of hypergammaglobulinemia because of HIV infection remains unclear 16. Here, we showed that 8 patients, including 1 patient who had hypergammaglobulinemia and thrombocytopenia, were diagnosed with HIV infection as a differential diagnosis of hypergammaglobulinemia. However, thrombocytopenia is present in 5–30% of HIV patients 17. Kaslow et al. demonstrated a higher prevalence of thrombocytopenia in patients with CD4 counts <200 cells/mm^3^ compared with those who had levels >700 cells/mm^3^ 18. In the current study, 7 patients underwent HIV testing during the differential diagnosis of thrombocytopenia, including 2 patients with pancytopenia; the CD4 counts in 4 of these 7 patients (57.1%) was <200 cells/mm^3^ at the time of diagnosing HIV infection. Although we could not clarify the prevalence of hypergammaglobulinemia and thrombocytopenia in all patients, these results indicate that abnormal blood test results were significantly associated with low CD4 counts.

The limitations of this study include the small study sample and the retrospective nature. In addition, we did not evaluate other factors that may affect HIV testing, including the city where the patients lived, their occupation and their family structure. Therefore, we could not determine whether the prognosis was affected by the reasons for HIV diagnosis and whether these results are applicable to other populations as well. However, we showed that voluntary HIV testing prevented late and very late diagnoses of HIV infection and that nonspecific abnormal blood test results, such as hypergammaglobulinemia and thrombocytopenia, were significantly associated with low CD4 counts of < 200/mm^3^. Therefore, we believe that voluntary HIV testing should be encouraged for early diagnosis and that physicians should lower their threshold for HIV screening to include all patients with symptoms or signs that may indicate an HIV infection.

## AUTHOR CONTRIBUTIONS

Hori S, Horino T Sato F, Kato T, Hosaka Y, Shimizu A, Kawano S, Hoshina T, Nakaharai K, Nakazawa Y, Yoshikawa K and Yoshida M contributed to collecting and confirming the data from medical records. Hori S and Horino T assessed and analyzed the data.

## Figures and Tables

**Table 1:** Characteristics of patients with HIV infection.

	Total		Late diagnosis		Non-late diagnosis		*p*-value	Very late diagnosis		Non-very-late diagnosis		*p*-value
	n=459		n=282		n=177			n=168		n=291		
	n	(%)	n	(%)	n	(%)		n	(%)	n	(%)	
Male gender	437	(95.2)	267	(94.7)	170	(96.0)	0.505	159	(94.6)	278	(94.9)	0.667
MSM	359	(82.2)	207	(77.5)	152	(89.4)	0.002	121	(76.1)	238	(85.0)	0.015
Age (median and range)	36	(18–71)	37	(20–70)	33	(18–71)	0.001	39	(20–68)	34	(18–71)	<0.001
The reason for HIV testing												
AIDS-defining illness	84	(18.3)	80	(28.4)	4	(2.3)	< 0.001	71	(42.3)	13	(4.5)	<0.001
Sexually transmitted infection	53	(11.5)	28	(9.9)	25	(14.1)	0.171	12	(7.1)	41	(14.1)	0.025
Nonspecific symptom	80	(17.4)	51	(18.1)	29	(16.4)	0.640	34	(20.2)	46	(15.8)	0.228
Nonspecific abnormal result	15	(3.3)	13	(4.6)	2	(1.1)	0.041	11	(6.5)	4	(1.4)	0.003
Routine testing	50	(10.9)	36	(12.8)	14	(7.9)	0.104	14	(8.3)	36	(12.4)	0.181
Voluntary testing	177	(38.6)	74	(26.2)	103	(58.2)	< 0.001	26	(15.5)	151	(51.9)	<0.001

**Table 2a:** Relative factors for late diagnosis in the logistic regression analysis.

	Adjusted odds ratio	95% CI	*p*-value
MSM	0.488	0.287–0.831	0.008
AIDS-defining illness	11.410	3.998–32.563	<0.001
Voluntary testing	0.418	0.274–0.638	<0.001

**Table 2b:** Relative factors for very late diagnosis in the logistic regression analysis.

	Adjusted odds ratio	95% CI	*p*-value
AIDS-defining illness	11.196	5.747–21.814	<0.001
Voluntary testing	0.353	0.210–0.593	<0.001
Nonspecific abnormal result	5.637	1.723–18.443	0.004

## References

[1] Palella FJ, Delaney KM, Moorman AC, Loveless MO, Fuhrer J, Satten GA (1998). Declining morbidity and mortality among patients with advanced human immunodeficiency virus infection. HIV Outpatient Study Investigators. N Engl J Med.

[2] Lohse N, Hansen AB, Pedersen G, Kronborg G, Gerstoft J, Sorensen HT (2007). Survival of persons with and without HIV infection in Denmark, 1995-2005. Ann Intern Med.

[3] Kitahata MM, Gange SJ, Abraham AG, Merriman B, Saag MS, Justice AC (2009). Effect of early versus deferred antiretroviral therapy for HIV on survival. N Engl J Med.

[4] Severe P, Juste MA, Ambroise A, Eliacin L, Marchand C, Apollon S (2010). Early versus standard antiretroviral therapy for HIV-infected adults in Haiti. N Engl J Med.

[5] Timing of HAART initiation and clinical outcomes in human immunodeficiency virus type 1 seroconverters (2011). Arch Intern Med. 10.1001/archinternmed.2011.401.

[6] Chadborn TR, Delpech VC, Sabin CA, Sinka K, Evans BG (2006). The late diagnosis and consequent short-term mortality of HIV-infected heterosexuals (England and Wales, 2000-2004). AIDS.

[7] Girardi E, Sabin CA, Monforte AD (2007). Late diagnosis of HIV infection: epidemiological features, consequences and strategies to encourage earlier testing. J Acquir Immune Defic Syndr.

[8] Lucas SB, Curtis H, Johnson MA (2008). National review of deaths among HIV-infected adults. Clin Med (Lond).

[9] Cohen MS, Chen YQ, McCauley M, Gamble T, Hosseinipour MC, Kumarasamy N (2011). Prevention of HIV-1 infection with early antiretroviral therapy. N Engl J Med.

[10] Marks G, Crepaz N, Senterfitt JW, Janssen RS (2005). Meta-analysis of high-risk sexual behavior in persons aware and unaware they are infected with HIV in the United States: implications for HIV prevention programs. J Acquir Immune Defic Syndr.

[11] Hall HI, Halverson J, Wilson DP, Suligoi B, Diez M, Le Vu S (2013). Late diagnosis and entry to care after diagnosis of human immunodeficiency virus infection: a country comparison. PloS One.

[12] Wohlgemut J, Lawes T, Laing RB (2012). Trends in missed presentations and late HIV diagnosis in a UK teaching hospital: a retrospective comparative cohort study. BMC Infect Dis.

[13] Antinori A, Coenen T, Costagiola D, Dedes N, Ellefson M, Gatell J (2011). Late presentation of HIV infection: a consensus definition. HIV Med.

[14] Daar ES, Little S, Pitt J, Santangelo J, Ho P, Harawa N (2001). Diagnosis of primary HIV-1 infection. Los Angeles County Primary HIV Infection Recruitment Network. Ann Intern Med.

[15] Amara S, Dezube BJ, Cooley TP, Pantanowitz L, Aboulafia DM (2006). HIV-associated monoclonal gammopathy: a retrospective analysis of 25 patients. Clin Infect Dis.

[16] Nagase H, Agematsu K, Kitano K, Takamoto M, Okubo Y, Komiyama A (2001). Mechanism of hypergammaglobulinemia by HIV infection: circulating memory B-cell reduction with plasmacytosis. Clin Immunol.

[17] Liebman HA (2008). Viral-associated immune thrombocytopenic purpura. Hematology Am Soc Hematol Educ Program.

[18] Kaslow RA, Phair JP, Friedman HB, Lyter D, Solomon RE, Dudley J (1987). Infection with the human immunodeficiency virus: clinical manifestations and their relationship to immune deficiency. A report from the Multicenter AIDS Cohort Study. Ann Intern Med.

